# Effectiveness of fall prevention interventions for community-dwelling adults aged 60 years and above in low- and middle-income countries: a systematic review and meta-analysis

**DOI:** 10.1186/s12877-026-07319-8

**Published:** 2026-03-18

**Authors:** Felicia Marian Dhananjee Chellapillai, Thusharika Dilrukshi Dissanayaka, Ishanka Weerasekara, Anne Tiedemann, Keith David Hill, Sobika Sivarasa, Abdul Majeed Mohamed Rikas, Dawake Leemagaskotuwe Gedara Heshani Madhuwanthi Samarasekara, Ziad Mohamed Fathima Zahra Safinaz, Anula Kariyawasam

**Affiliations:** 1 Department of Physiotherapy, Faculty of Allied Health Sciences, General Sir John Kotelawala Defence University, Werahera, Colombo, Sri Lanka; 2 Department of Physiology, Faculty of Medicine, University of Peradeniya, Peradeniya, Sri Lanka; 3 Department of Physiotherapy, Faculty of Medicine, Nursing and Allied Health Sciences, Monash University, Melbourne, Australia; 4 Institute of Health and Wellbeing, Federation University, Churchill, Victoria Australia; 5 School of Allied Health Science and Practice, Faculty of Health and Medical Sciences, The University of Adelaide, Adelaide, Australia; 6 Faculty of Medicine and Health, School of Public Health Gadigal Country, The University of Sydney, Sydney, Australia; 7 Rehabilitation, Ageing and Independent Living (RAIL) Research Centre, School of Primary and Allied Health Care, Peninsula Campus, Monash University, Melbourne, Australia; 8 Department of Nursing, Faculty of Allied Health Sciences, University of Jaffna, Jaffna, Sri Lanka; 9 Department of Physiotherapy, Faculty of Allied Health Sciences, University of Peradeniya, Peradeniya, Sri Lanka; 10 Healing First Physiotherapy, Kandy, Sri Lanka; 11 Ayati National Centre for Children with Disabilities, Ragama, Western Province Sri Lanka

**Keywords:** Accidental falls, Community-dwelling, Older adults, Low- and middle-income countries

## Abstract

**Background:**

Falls are a major cause of disability and mortality in older age. There is clear evidence of effective fall prevention interventions that have informed global guidelines, however much of the evidence comes from high-income countries. The objective of this study was to identify and assess the effectiveness of fall prevention interventions for community-dwelling older people living in low- and middle-income countries (LMICs).

**Methods:**

Studies published up to March 2025, in Medline, Embase, the Cochrane Library, Scopus, CINAHL and references of previous reviews were searched. A study was included if an intervention was used to prevent falls or to improve fall-related outcomes among community-dwelling older adults in LMICs. Titles, abstracts, full texts and study quality were screened by two independent reviewers and conflicts were resolved by a third reviewer. Data were extracted by two independent reviewers and studies which had sufficient data were included for meta-analysis.

**Results:**

Among the retrieved 2013 studies, 30 relevant studies with 9817 participants (65% female) from 12 LMICs were included. The participants’ ages ranged from 60 to 94 years. Nine studies were eligible for meta-analysis and only two to four studies were able to be pooled for each outcome. According to the meta-analysis, exercise significantly improved balance (standardised mean difference (SMD) = 1.18, [0.30,2.06], *p* = 0.008), while it had no effect on falls (SMD=-0.4, [-1.08,0.29], *p* = 0.18) and fall-related outcomes such as mobility (SMD=-0.50, [-1.13,0.12], *p* = 0.11), and fear of falling (SMD=-0.04, [-0.69,0.61], *p* = 0.90). According to the narrative synthesis interventions such as exercise, dance, and education programs may reduce falls and fall-related outcomes.

**Conclusion:**

Exercise, a senior dance program, Thai traditional dance, and education programs were among the interventions identified in the included studies. Exercises could improve balance in community-dwelling older adults living in the LMICs. However, the effectiveness of these interventions in reducing falls or other fall-related outcomes is uncertain due to the limited number of studies with small samples available from LMICs.

**Trial registration:**

CRD42022335448, 07th June, 2022.

**Supplementary Information:**

The online version contains supplementary material available at 10.1186/s12877-026-07319-8.

## Introduction

Falls are a major health issue among older adults, which negatively impact independence and quality of life [1]. The global prevalence of falls in community dwelling older adults is 29.8%, while the percentage of prevalence in High-Income Countries is 30% [2, 3]. The prevalence of falls among community dwelling older adults in Low- and Middle-Income countries (LMICs) such as Ethiopia, India, Nigeria, South Africa and Sri Lanka are 28.4%, 37.5%, 25.3%, 26.4% and 34.3% respectively [4–8]. Although fall rate and fall risk is not always higher in the LMICs, the outcomes of falls, such as fall related injury, disabilities or deaths may be severe in LMICs when compared to high-income countries. This is mainly due to limited access to emergency care and rehabilitation services [9, 10]. Impaired balance and strength, fear of falling (FOF), environmental hazards, impaired vision and polypharmacy are some common risk factors for falls among older adults [11, 12]. Falls commonly lead to injuries, loss of independence, functional decline, loss of self-confidence, social isolation, depression, and even death [13, 14]. The consequences of falls often demand significant economic and social support for treatment and rehabilitation. Therefore, considering the growing proportion of older people across the globe, widespread implementation of effective fall prevention interventions is crucial for supporting healthy ageing.

There is now clear evidence about effective strategies to prevent falls [15]. The Prevention of Falls Network Europe (ProFaNE) categorises fall prevention interventions as single, multiple-component and multifactorial interventions [16]. Exercise is one of the most commonly tested single interventions found to be effective in preventing falls among community-dwelling older adults [15, 17, 18]. Multiple-component interventions may include two or more fixed fall prevention interventions provided to all participants, while in multifactorial interventions, fall prevention interventions are prescribed on an individual basis according to the specific identified risk factors [16].

Most studies that have explored the effectiveness of fall prevention interventions are from high-income countries. A 2019 systematic review on effective exercise for fall prevention included 108 randomised controlled trials (RCTs) from 25 countries [17]. Among these studies only 16 RCTs (15%) were from LMICs and the rest were from high income countries. According to this review, exercise is an effective intervention to prevent falls. But as this review consisted of mostly studies from high-income countries the results may not generalise to LMICs. Due to the difference in economic status and other factors such as culture, health systems, lifestyle, family role, indoor and outdoor environments, and type of footwear, the effects of existing fall prevention interventions in the LMIC context might be different. Therefore, it is important to explore the current practice for fall prevention in LMICs [19–21].

Even though the World Guidelines for Falls Prevention and Management were primarily developed based on evidence from HICs, they have also highlighted the challenges related to implementation in LMICs. However, these guidelines do not provide separate recommendations specifically for LMICs. Instead, they emphasize that while the core components of screening, assessment, and interventions are universal, their implementation should be adapted according to the local context, available resources, and healthcare system capacity [15].

The objective of this study was to identify and assess the effectiveness of fall prevention interventions on fall rate and fall related outcomes including fall risk, fear of falling, balance and mobility among community-dwelling older people living in LMICs.

## Methods

### Protocol registration

Preferred Reporting Items for Systematic Reviews and Meta-analysis (PRISMA) guidelines guided the conduct and reporting of this systematic review [22, 23]. The protocol for this systematic review and meta-analysis was prospectively registered in the International prospective register of systematic reviews (PROSPERO, Registration number: CRD42022335448, 07th June, 2022).

### Search strategy

Medline, Embase, Cochrane, Scopus, CINAHL and references of previous reviews which investigated fall prevention practices in any country. were searched to identify relevant studies published up to March 21st, 2025, for quantitative studies, which focused on fall prevention interventions for community-dwelling older adults from LMICs [24–29]. Countries with low- and middle-income economies were defined based on the Organization for Economic Co-operation and Development (OECD) definition, which considers the gross national income (GNI) per capita according to the World Bank, in U.S. dollars., and classifies the countries as low-income countries (USD1005 or less), lower-middle-income countries (USD1006-USD3955) and upper-middle-income countries (USD3956-USD12235) [30]. The search strategy is included as a supplementary file.

### Selection criteria

A study was included if: (1) the participants were aged 60 years and above, and living in the community, (2) an intervention was given to prevent falls or to address fall-related outcomes, (3) the study was conducted in one or more LMICs, and (4) study designs using quantitative methods were used. Only studies published in English were included in this review. Studies were excluded if they did not align with the inclusion criteria.

Titles and abstracts were imported to the Covidence systematic review tool and screened by two independent reviewers (DC and SS) to identify potentially relevant studies [31]. The full text of the selected studies was then independently reviewed by two reviewers (DC and SS). Disagreements were resolved by a third author (TDD).

### Outcome measures

The main outcome of interest was the rate of falls. The additional outcomes were risk of falls, fear of falls, number of fallers, fall-related fractures, fall related hospital admissions, as well as physical performance measures, including balance, mobility, lower limb strength, aerobic fitness, gait speed, and endurance.

### Data extraction

A table was created to extract descriptive data such as authors, published year, study design, country, sample size, population, parameters of the intervention and control, inclusion and exclusion criteria, outcome measures, and the effect of intervention. The studies were categorized according to their study design. When an outcome was measured using different tools, the data were extracted separately according to the tools used. When enough information was not available, the corresponding authors were contacted to receive additional information. The data were extracted by any two authors (DC, RM, SS, ZS, and HS).

### Quality assessment

Any two review authors (DC, RM, SS, ZS, and HS) independently assessed the Risk of Bias (ROB) of the included studies using the Joanna Briggs Institute (JBI) critical appraisal tools [32]. The JBI tools developed individually for RCTs, quasi-experimental studies, analytical cross-sectional studies and cohort studies were used to assess the quality of the studies according to their study designs [33–36]. Any disagreements were resolved with a third reviewer (TDD).

### Data analysis

#### Meta-analyses

Results were pooled using RevMan 5 software (version 5.2) [37]. Studies were pooled for meta-analysis if at least two studies with the same study design evaluated the same intervention for the same outcome using the same assessment tools. Accordingly, eight studies were able to be included in the meta-analyses. However, only two to four studies could be pooled for each outcome category. We assumed that clinical and methodological heterogeneity was likely to exist in the included studies, and therefore, we used random effects meta-analyses [38]. To account for differences in outcome measures between trials, we calculated standardised mean differences to measure the effect size. Heterogeneity was interpreted based on I² values as follows: 0–40% might not be important, 30%-60% may represent moderate heterogeneity, 50–90% may represent substantial heterogeneity and 75%-90% was considered considerable heterogeneity [39].

To evaluate the robustness of the results, leave–one–out analyses were performed by excluding each study in turn to expose the potential influence of the individual studies on the pooled estimate. The funnel plot to assess the publication bias was not developed since the meta-analysis included less than ten studies [40].

#### Narrative synthesis

The RCTs, quasi-experimental studies, cohort study, and the cross-sectional study which were not included for the meta-analysis were considered for the narrative synthesis.

## Results

### Description of studies

**Fig. 1:**
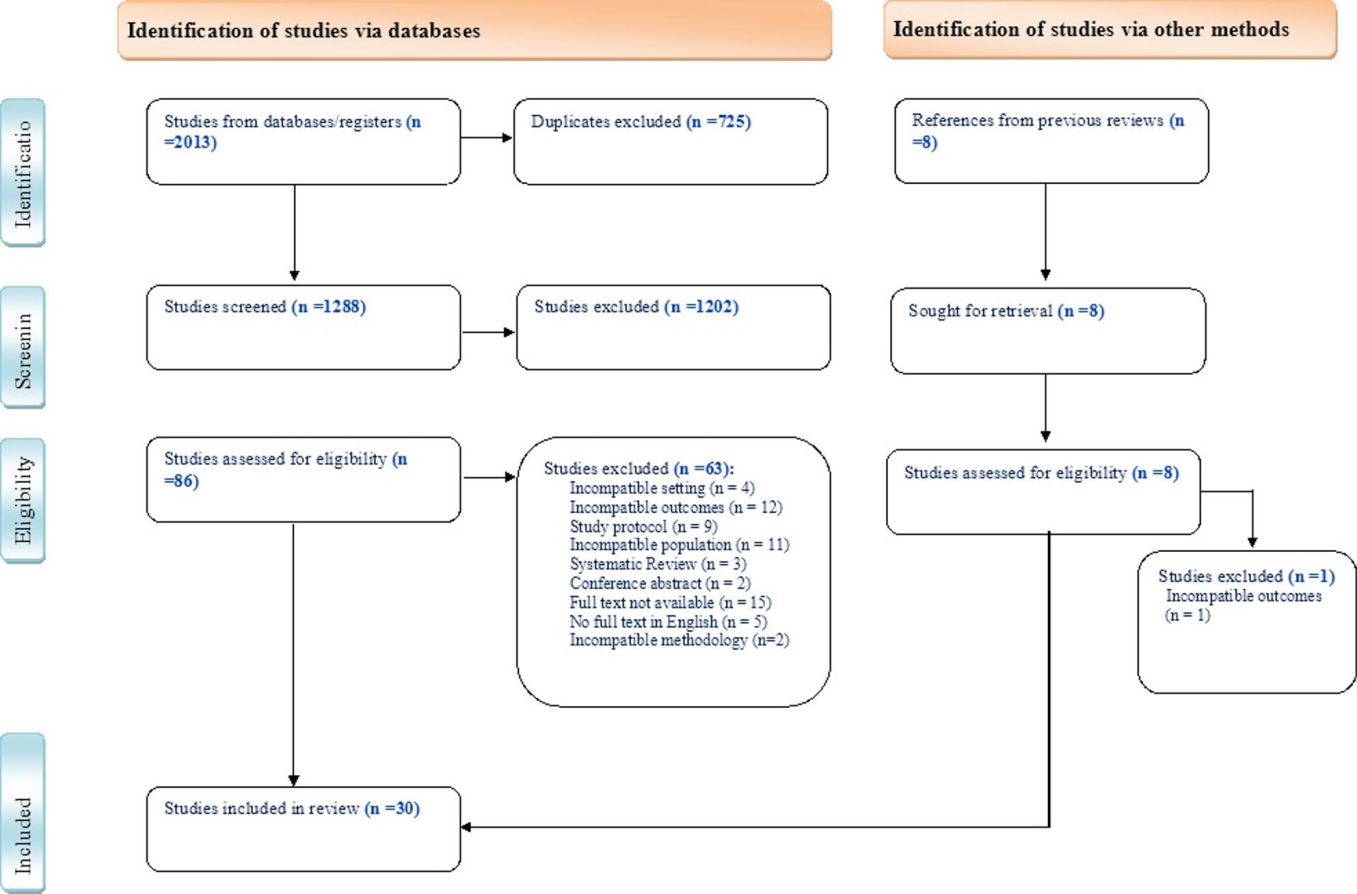
Identification, screening, eligibility and inclusion of studies

As shown in Fig. 1, 2013 studies were retrieved after excluding the duplicates (725). Among those, 30 studies were included for this review (*n* = 9817) and nine studies were included for meta-analysis. The remaining 21 studies were only analysed narratively.

### Study characteristics

Altogether, 9817 participants from 30 studies were included in this review. The age of the participants in the included studies ranged from 60 to 94 years. 65% of the participants were females. The details of the studies are included in Table 1. Among the 30 studies there were 21 RCTs, seven quasi-experimental studies, one cross-sectional study and one cohort study. The sample size within studies ranged from 25 to 2799 (327.23 ± 641.05). The studies assessed the effectiveness of certain interventions on either minimizing falls, fear of falling and other fall related outcomes or improving balance, mobility, lower limb strength, endurance, and/or aerobic fitness which may directly or indirectly prevent falls.

**Table 1:** Characteristics of studies included in the review

Author year	Country	Study design or method				Sample size	Sex M/F	Age
		RCT	Cohort	Cross-sectional	Quasi			
Almeida et al., 2013 [41]	Brazil	✓				76	13/63	70–89
Altamirano et al., 2022 [42]	Ecuador	✓				378	93/285	65–94
Arantes et al., 2015 [43]	Brazil	✓				30	0/30	65–81
Aranyavalai et al., 2020 [44]	Thailand		✓			255	72/183	60–89
Arghavani et al., 2019 [45]	Iran				✓	40	40/0	66–73
Assantachai et al., 2002 [46]	Thailand				✓	1043	472/581	61–74
Boongird et al., 2017 [47]	Thailand	✓				439	77/362	60–88
Bunout et al., 2005 [48]	Chile	✓				298	87/211	70–80
Dadgari et al., 2016 [49]	Iran	✓				317	NG	65–76
Dangour et al., 2011 [50]	Chile	✓				2799	906/1893	65–68
Franco et al., 2019 [51]	Brazil	✓				82	17/65	62–75
Guerra et al., 2021 [52]	Brazil	✓				118	39/79	65–75
Hall et al., 2010 [53]	Georgia				✓	49	25/24	68–83
Ing et al., 2024 [54]	Malaysia	✓				52	8/44	61–76
Irez et al., 2011 [55]	Turkey				✓	60	0/60	66–84
Kocaman et al., 2021 [56]	Turkey	✓				30	14/16	69–86
Kraiwong et al., 2021 [57]	Thailand	✓				37	8/29	60–86
Kulkarni et al., 2023 [58]	India				✓	181	85/96	65–73
Lipardo et al., 2020 [59]	Phillippines	✓				92	19/73	60–83
Madureira et al., 2010 [60]	Brazil	✓				60	0/60	64–84
Mohammadi et al., 2024 [61]	Iran	✓				126	28/98	61–66
Noopud et al., 2019 [62]	Thailand	✓				43	0/43	60–80
SanjuánVásquez et al., 2019 [63]	Mexico				✓	25	1/24	60–70
Silva et al., 2013 [64]	Brazil			✓		85	18/67	64–80
Sungkarat et al., 2017 [65]	Thailand	✓				66	9/57	60–75
Suttanon et al., 2018 [66]	Thailand	✓				277	74/203	66–78
Stonsaovapak et al., 2022 [67]	Thailand				✓	121	18/103	61–73
Tan et al. 2018 [68]	Malaysia	✓				268	92/176	61–90
Xia et al., 2009 [69]	China	✓				2310	1097/1213	64–80
Yu et al., 2025 [70]	China	✓				60	30/30	65–79

*Abbreviations: RCT *Randomised Controlled Trial, *Quasi *Quasi-experimental studies, * M/F *Male/Female*, NG *Not given

Among the included studies there were four single-arm studies (cohort *n* = 1, quasi-experimental *n* = 3), 20 double-arm studies (RCT *n* = 15, quasi-experimental studies *n* = 4, cross-sectional studies *n* = 1), four three-arm studies (RCT *n* = 4), and two four-arm study (RCT *n* = 2). The interventions were either standalone (*n* = 24) or multicomponent interventions (*n* = 6). The interventions used in these studies were walking (*n* = 2), postural training (*n* = 2), education (*n* = 4), exercises (*n* = 22), dance (*n* = 3), cognitive training (*n* = 2), multisensory training (*n* = 1), environmental intervention (*n* = 2), nutrition supplements (*n* = 1) and Russian stimulation (an electrical stimulation which induces skeletal muscle contraction) (*n* = 1). Table 2 shows the intervention and control groups used in RCTs and quasi-experimental studies.

There were a variety of exercise types prescribed in these studies, including balance training (*n* = 9), resistance training (*n* = 8), gait training (*n* = 3), flexibility (*n* = 4), endurance (*n* = 1), neuro-motor exercises (*n* = 1), aerobic exercises (*n* = 2), vestibular exercise (*n* = 1) and square stepping exercise (*n* = 1). Three studies included traditional interventions such as Tai Chi (*n* = 1), Baduanjin (*n* = 1) and Thai traditional dance (*n* = 1). One study used a senior dance program as the intervention. Among the studies which included exercise as a single intervention or a component of their intervention, four were multimodal exercise programs. This included at least two (maximum *n* = 4) of the above-mentioned exercise types combined within a single program. The age of participants ranged from 60 to 94 years. Among the studies, one study included only males, four studies included only females, while the rest (*n* = 25) included both males and females.

### Risk of bias assessment

The risk of bias (ROB) assessment of the included studies is shown in Table 3. Most studies were rated as high quality. In most of the RCTs participants and the treatment providers were not blinded, but the outcome assessors were blinded. The participants in both intervention and control group were similar at baseline and both groups were treated identically other than the treatment of interest in most of the RCTs. The quasi-experimental studies, cohort study and the cross-sectional study were of good quality according to the ROB assessments.

**Table 2:** Intervention and control groups used in RCTs and quasi-experimental studies

Study	Country	Study design	Intervention	Control	Duration	Frequency	Outcome measures	Effect of the intervention
Almeida et al., 2013 [41]	Brazil	Three- armed RCT	Group 1: Fully supervised exercise group Group 2: Minimally supervised exercise group	No intervention	12 weeks	Three times a week	Balance, Functional mobility, Endurance (400 m walk test)	Endurance Significant improvement in group 1 Functional mobility Significant improvement in group 1 and 2 Balance Significant improvement in group 1
Altamirano et al., 2022 [42]	Ecuador	Two level cluster RCT	Supervised physical training program including strength, balance and gait training.	Usual care	12 months	1 h per week	Fall incidence, fear of falling, functional mobility, fall related injuries, lower body strength, balance	Fall incidence Significantly less Fear of falling Significant reduction Functional mobility Significant improvement Fall related injuries Significant reduction Lower body strength No significant improvement Balance Significant improvement
Arantes et al., 2015 [43]	Brazil	Double-armed RCT	Balance exercise program	Non-balance-focused exercise program	12 weeks	Twice a week	Balance, functional mobility, fear of falling, gait speed	Fear of falling Significant reduction Balance Significant improvement Functional mobility Significant improvement Gait speed Significant improvement
Boongird et al.,2017 [47]	Thailand	Double-armed RCT	Home-based exercise program (lower extremity strengthening, stretching and balance training).	Fall prevention education	12 months	120 min per week	Fall rate, fear of falling, mobility, balance	Fall rate No significant reduction Fear of falling Significant reduction Mobility No significant improvement Balance No significant improvement
Bunout et al., 2005 [48]	Chile	Double-armed RCT	Moderate-intensity resistance training	No structured exercise program	12 months	Twice a week, one hour each	Body composition, gait speed, grip strength, quadriceps and biceps strength, fall incidence, cognitive function	Body composition No significant improvement Gait speed Significant improvement Grip strength No significant improvement Quadriceps strength Significant improvement Biceps strength Significant improvement Fall incidence No significant improvement Cognitive function No significant improvement
Dadgari et al., 2016 [49]	Iran	Double-armed RCT	Otago Home-based Exercise Program	General health training	6 months	Three times a week 45–60 min each	Balance, fall incidence, mobility, Upper extremity strength, lower limb strength	Balance Significant improvement Fall incidence Significant reduction Mobility Significant improvement Upper extremity strength Significant improvement Lower limb strength Significant improvement
Dangour et al., 2011 [50]	Chile	2 × 2 factorial cluster RCT	Group 1: Micronutrient-rich nutritional supplement Group 2: Physical activity Group 3: Micronutrient-rich nutritional supplement with physical activity	No intervention	24 months	Daily nutrition supplement and physical activity twice a week	Incidence of pneumonia, functional mobility, endurance, body composition, depression, falls incidence, fractures, blood pressure	Incidence of pneumonia No significant improvement Functional mobility Significant improvement in group 2 and 3 Endurance Significant improvement in group 2 and 3 Body composition No significant improvement Depression No significant improvement Falls incidence No significant improvement Fractures No significant improvement Blood pressure No significant improvement
Franco et al., 2019 [51]	Brazil	Double-armed RCT	Falls prevention education plus senior dance program	Falls prevention education only	12 weeks	60 min sessions, twice weekly	Single leg stance, sit-to-stand test, 4-meter walk test.	Single leg stance Significant improvement sit-to-stand test Significant improvement 4-meter walk test. Significant improvement
Guerra et al., 2021 [52]	Brazil	Double-armed RCT	Fall prevention education	Usual care	12 weeks	Two home visits within 3 months	Fall incidence, fear of falling, fall related injury	Fall incidence Significantly less Fear of falling Significant reduction Fall related injury Significantly less
Ing et al., 2024 [54]	Malaysia	Double-armed RCT	Virtual supervised group-based fall prevention exercise	One session of virtual fall prevention education	24 weeks	Twice a week, 75–80 min per session	Mobility, balance, lower limb muscle strength, handgrip strength, quality of life, fear of falling	Mobility Significant improvement Balance Significant improvement Lower limb muscle strength Significant improvement Handgrip strength Significant improvement Quality of life No significant improvement Fear of falling No significant improvement
Kocaman et al., 2021 [56]	Turkey	Three-armed RCT	Group 1: Posturography balance exercise plus vestibular exercise Group 2: Square step exercise plus vestibular exercise	Group 3: Vestibular exercise only	8 weeks	Three times a week	Balance, fear of falling, cognitive functions, activities of daily living, quality of life	Balance Significant improvement in group 1 and 2 Fear of falling Significant reduction in all groups Quality of life Significant improvement in all groups Cognitive functions Significant improvement in group 1 and 2 Activities of daily living Significant improvement in group 1 and 2
Kraiwong et al., 2021 [57]	Thailand	Double-armed RCT	Group-based physical-cognitive training	Health education	8 weeks	Three times a week 45–60 min per session	Functional mobility, coordination, hip extensor strength, hip flexor strength, hip abductor strength, Knee extensor strength, Knee flexor strength, ankle plantarflexor strength, ankle dorsiflexor strength, Barthel index, cognitive function, fall incidence, fear of falling	Functional mobility Significant improvement Coordination Significant improvement Hip extensor strength Significant improvement Hip flexor strength No Significant improvement Hip abductor strength Significant improvement Knee extensor strength Significant improvement Knee flexor strength Significant improvement Ankle plantarflexor strength Significant improvement ankle dorsiflexor strength Significant improvement Barthel index Significant improvement Cognitive function No Significant improvement Fall incidence No Significant improvement Fear of falling No Significant improvement
Lipardo et al., 2020 [59]	Philippines	Four-armed RCT	Group 1: Physical training only Group 2: Cognitive training only Group 3: Combined physical and cognitive training	No intervention	12 weeks	Three times a week for 60–90 min	Fall incidence, overall fall risk, dynamic balance, gait speed and lower limb strength	Fall incidence No significant difference Overall fall risk No significant difference Dynamic balance Significant improvement in group 2 and 3 Gait speed No significant difference Lower limb strength No significant difference
Madureira et al., 2010 [60]	Brazil	Double-armed RCT	Balance training program	No intervention	12 months	Three times per week.	Quality of life, functional balance and fall rate	Quality of life Significant improvement Functional balance Significant improvement Fall rate Significant reduction
Mohammadi et al., 2024 [61]	Iran	Three-armed RCT	Group 1: Otago home-based exercise program Group 2: Chair squat exercise	No intervention	8 weeks	Three times a week for 45 min	Fear of falling and quality of life	Fear of Falling Significant reduction in group 1 and 2, while significantly higher effect in group 1 Quality of life Significantly improved in groups 1 and 2
Noopud et al., 2019 [62]	Thailand	Double-armed RCT	Thai traditional dance	No intervention	12 weeks	Twice a week 30–60 min	Balance, mobility, postural control, agility, gait speed.	Balance Significantly improved Mobility Significantly improved Postural control Significantly improved Agility Significantly improved Gait speed Significantly improved
Sungkarat et al., 2017 [65]	Thailand	Double-armed RCT	Tai Chi	No intervention	15 weeks (3 weeks centre based and 12 weeks at home)	Three times a week for 30 min.	Cognitive status, fall risk	Cognitive status Significantly improved Fall risk Significantly improved
Suttanon et al., 2018 [66]	Thailand	Double-armed RCT	Balance training	No intervention	4 months	Four days a week	Falls rate, functional reach test, step test, 5 times sit to stand, TUG, physical activity level assessment, fear of falling	Falls rate No significant difference Functional reach test No significant difference Step test No significant difference 5 times sit to stand No significant difference TUG No significant difference Physical activity level assessment No significant difference Fear of falling No significant difference
Xia et al., 2009 [69]	China	Double-armed RCT	Multifaceted intervention (education programme, brochure distribution, poster exhibition, consultation and indoor and community safety through hazard assessment and hazard elimination)	No intervention	18 months	Education and consultations once in 2 months, home hazard assessment twice a year, poster and brochure distribution throughout the duration.	Fall rate	Fall rate Significant reduction
Tan et al., 2018 [68]	Malaysia	Double-armed RCT	Multifactorial intervention with modified OTAGO exercises, visual screening, home modifications, medication review, cardiovascular intervention and falls education	General health advice	12 weeks	Modified OTAGO for 4 times in 3 months.	Fall rate, Fall recurrence	Fall rate No significant difference Fall recurrence No significant difference
Yu et al., 2025 [70]	China	Three- armed RCT	Group 1: Baduanjin exercises Group 2: Brisk walking	No intervention	16 weeks	Four sessions per week. (Group 1: 70–90 min, Group 2: 35–45 min)	Static balance, body sway parameters, centre of pressure (COP) displacement	Static balance Significant improvement in group 1 Body sway parameters Significant improvement in group 1 centre of pressure (COP) displacement Significant improvement in group 1
Arghavani et al., 2019 [45]	Iran	Double-armed quasi-experiment study	Anticipatory postural adjustments focused training	No intervention	5 months	Three times a week for 60 min	Balance confidence, fear of falling, quality of life	Balance Confidence Significant improvement Fear of falling Significant reduction Quality of life Significant improvement
Assantachai et al., 2002 [46]	Thailand	Double-armed quasi-experiment study	Falls prevention education	No intervention	12 months		Incidence of falls	Incidence of falls Significant reduction
Hall et al., 2010 [53]	Georgia	Single-armed quasi-experiment study	Balance and gait training, and home exercise program	No control group	4 weeks	Three times a week	Balance confidence, gait speed, fall risk, sensory organization test, dual task ability	Balance confidence Significant improvement Gait speed Significant improvement Fall risk Significant reduction Sensory organization test Significant improvement Dual task ability Significant improvement
Irez et al., 2011 [55]	Turkey	Double-armed quasi-experiment study	Pilates-based exercise program	No intervention	12 weeks	Three times a week (1 h each)	Dynamic balance, flexibility, reaction time, falls, muscle strength	Dynamic balance Significant improvement Flexibility Significant improvement Reaction time Significant reduction Falls Significant reduction Muscle strength Significant improvement
Kulkarni et al., 2023 [58]	India	Double-armed quasi-experiment study	Video assisted falls prevention exercise program	No intervention	12 months	Five to seven times	incidence of falls, fear of falling, difficulty in ADL	Incidence of falls Significant reduction Fear of falling No significant difference Difficulty in ADL Significant reduction
SanjuánVásquez et al., 2019 [63]	Mexico	Single-armed quasi-experiment study	Russian stimulation with isometric contraction	No control group	12 weeks	Three times a week, for 10 min	Muscle strength of the quadriceps and tibialis anterior muscles, Berg balance score, Tinnetti mobility test, Get Up and Go Test	Quadriceps and tibialis anterior muscles Significant improvement Berg balance score Significant improvement Tinnetti mobility test Significant improvement Get up and go test Significant improvement
Stonsaovapak et al., 2022 [67]	Thailand	Single-armed quasi-experiment study	Tele-rehabilitation	No control group	08 weeks	Three times a week for 30 min	Short Physical Performance Battery, Timed up and go test, 6 min walk test	Short Physical Performance Battery Significant improvement Timed up and go test Significant improvement 6 min walk test Significant improvement

### Results from the meta-analysis

Altogether, 13 studies were eligible for meta-analyses, comprising four outcomes: fall incidence, FOF, mobility and balance; however, due to the unavailability of data from the published manuscripts and unsuccessful attempts to obtain missing data from the authors (*n* = 4) [42, 47, 49, 57], only nine studies were included for the meta-analyses [41, 43, 54–56, 58–60, 66, 67]. The remaining studies were summarised narratively to provide a comprehensive overview of fall prevention interventions in LMICs.

### Effect of exercise on falls

A sub-group analysis was conducted for two RCTs and two quasi-experimental studies with a total of 299 participants [55, 58, 60, 66]. Both the overall analysis and the sub-group analysis indicated that exercise had no effect on number of falls among community-dwelling older adults in LMICs (SMD = -0.4, CI 95% = -1.08, 0.29 *p* = 0.18, Fig. 2.1).

**Fig. 2:**
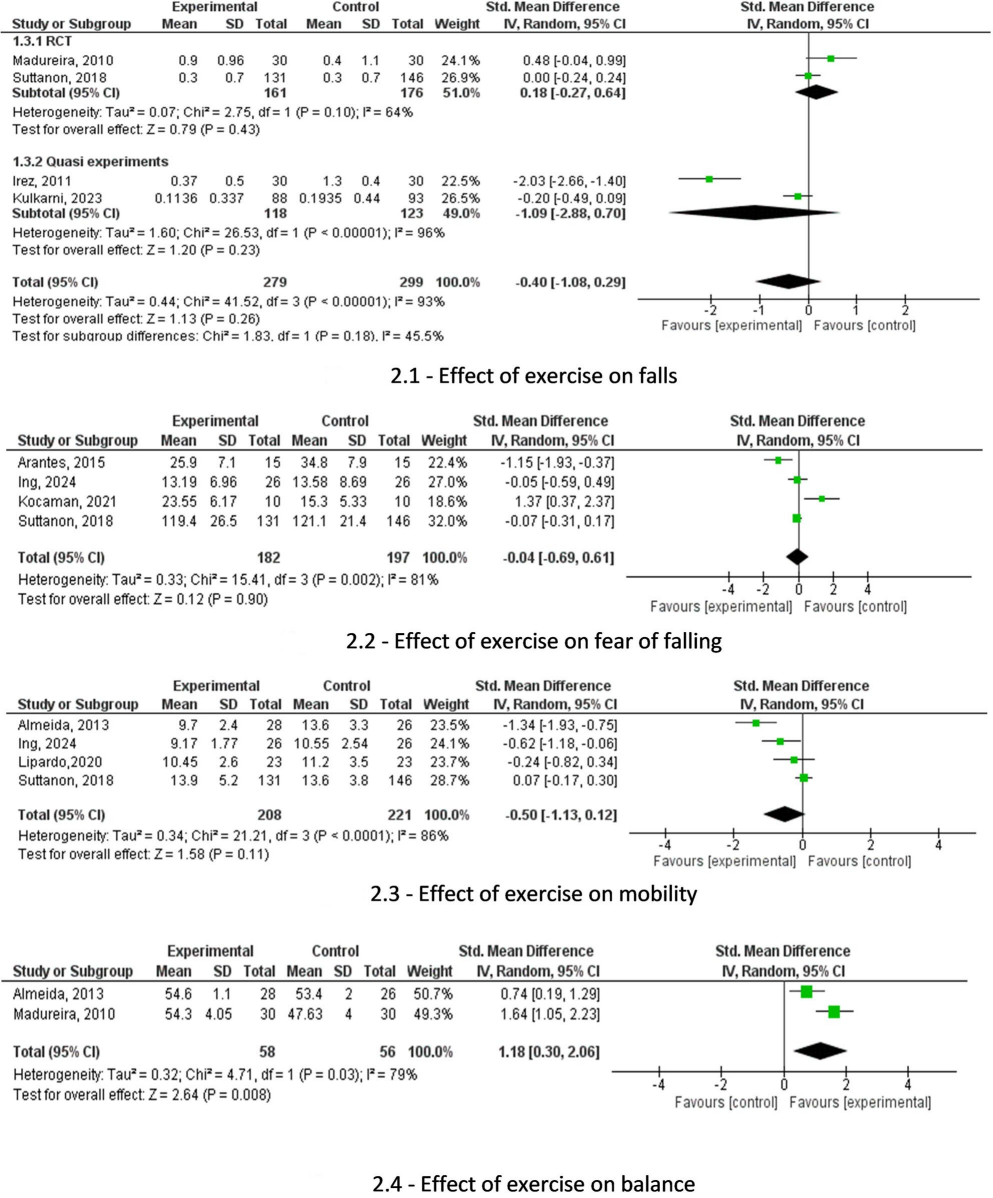
Results from the meta-analyses

### Effect of exercise on fear of falling

Four studies comprising a total of 197 participants were pooled to evaluate the effect of exercise on FOF [43, 54, 56, 66]. These studies used the Falls Efficacy Scale International (FESI) to assess fear of falling [71]. The results indicated that exercise had no effect on FOF (SMD = -0.04, 95% CI = -0.69 to 0.61, *p* = 0.90, Fig. 2.2).

### Effect of exercise on mobility

Four studies provided data to evaluate the effect of exercise on mobility assessed using the Timed-Up and Go (TUG) test [72]. There was a total of 271 participants [41, 54, 59, 66]. The results showed that there was no effect of exercise on mobility (SMD= -0.5, CI 95% = -1.13, 0.12, *p* = 0.11, Fig. 2.3).

### Effect of exercise on balance

Two studies provided data to evaluate the effect of exercise on balance assessed using the Berg balance scale [73]. There was a total of 114 participants [41, 60]. The results showed that exercises significantly improved balance (SMD = 1.18, CI 95%= 0.30, 2.06, *p* = 0.008, Fig. 2.4).

### Narrative synthesis

The purpose of this narrative synthesis is to summarize and interpret the findings of studies that examined interventions in reducing falls and improving fall-related outcomes among community-dwelling older adults in LMICs that were not able to be included in the meta-analyses. It presents findings from 14 RCTs, five quasi-experimental studies, one cross-sectional study and one cohort study [44–47, 49, 51, 53, 61–65, 67, 69, 70]. These studies were excluded from the meta-analysis due to data unavailability or lack of directly comparable outcomes. Instead, a narrative synthesis was conducted to explore the reported direction and magnitude of intervention effects and/or implications for fall prevention implementation among community-dwelling older adults in LMICs. The details of the included studies are given in Tables 1 and 2.

**Table 3:** Risk of Bias assessment of recruited studies

**ROB of RCTs**													
	**True randomization**	**Concealed allocation**	**Similarity between treatment groups**	**Blinded participants**	**Blinded treatment providers**	**Blinded outcome assessors**	**Identical treatment of groups**	**Follow up complete**	**Uniform outcome measurements in groups**	**Participants analysed in the groups to which they were randomised**	**Outcomes measured in a reliable way**	**Appropriate statistical analysis**	**Appropriate trial design**
Almeida et al., 2013 [41]	△	❍	△	□	□	❍	△	□	△	△	△	△	△
Altamirano et al., 2022 [42]	△	❍	△	□	□	❍	△	□	△	△	△	△	△
Arantes et al.,2015 [43]	△	❍	△	❍	❍	△	△	△	△	△	△	△	△
Boongird et al., 2017 [47]	△	△	△	□	□	△	△	□	△	△	△	△	△
Bunout et al., 2005 [48]	△	❍	△	□	□	❍	△	□	△	△	△	△	△
Dadgari et al., 2016 [49]	△	△	△	❍	❍	△	△	❍	△	△	△	△	△
Dangour et al., 2011 [50]	△	□	△	□	□	△	△	□	△	△	△	△	△
Franco et al., 2019 [51]	△	△	△	□	□	△	△	□	△	△	△	△	△
Guerra et al., 2021 [52]	△	△	△	□	□	△	△	□	△	△	△	△	△
Ing et al., 2024 [54]	△	△	△	□	□	△	△	❍	△	△	△	△	△
Kocaman et al., 2021 [56]	△	❍	△	❍	❍	❍	△	❍	△	△	△	△	△
Kraiwong et al., 2021 [57]	△	❍	△	❍	❍	△	△	□	△	△	△	△	△
Lipardo et al., 2020 [59]	△	△	△	□	□	△	△	□	△	△	△	△	△
Madureira et al., 2010 [60]	△	△	△	□	□	△	△	□	△	△	△	△	△
Mohammadi et al., 2024 [61]	△	△	△	□	□	△	△	□	△	△	△	△	△
Noopud et al., 2019 [62]	△	❍	△	❍	❍	△	△	□	△	△	△	△	△
Sungkarat et al., 2017 [65]	△	△	△	□	□	△	△	□	△	△	△	△	△
Suttanon et al., 2018 [66]	△	△	△	□	□	△	△	□	△	△	△	△	△
Tan et al., 2018 [68]	△	△	△	□	□	□	△	□	△	△	△	△	△
Xia et al., 2009 [69]	△	△	△	□	□	△	△	❍	△	△	△	△	△
Yu et al., 2025 [70]	△	△	△	□	□	△	△	❍	△	△	△	△	△
**ROB of Quasi-experimental studies**													
	**Clear cause and effect of the study**	**Similar participants included for any comparisons**		**Identical treatment of groups**	**Had a control group**	**Multiple measurements of the outcome both pre and post the intervention/exposure**		**Follow up complete**		**Uniform outcome measurements in groups**	**Outcomes measured in reliable way**	**Appropriate statistical analysis was used**	
Arghavani et al., 2019 [45]	△	△		❍	△	△		□		△	△	△	
Assantachai et al., 2002 [46]	△	△		△	△	△		❍		△	△	△	
Hall et al., 2010 [53]	△	□		□	□	△		△		❍	△	△	
Irez et al.,2011 [55]	△	△		❍	△	△		❍		△	△	△	
Kulkarni et al., 2023 [58]	△	△		△	△	△		□		△	△	△	
SanjuánVásquez et al., 2019 [63]	△	□		□	□	△		△		△	△	△	
Stonsaovapak et al., 2022 [67]	△	□		□	□	△		□		△	△	△	
**ROB of cross-sectional studies**													
	**Clear Inclusion criteria is clearly defined**	**Study subjects and the setting described in detail**		**The exposure measured in a valid and reliable way**		**Standard criteria used for measurement of the condition**		**Confounding factors identified**		**Strategies to deal with confounding factors were stated**	**Outcomes measured in a valid and reliable way**	**Appropriate statistical analysis used**	
Silva et al., 2013 [64]	△	△		△		△		❍		❍	△	△	
**ROB of cohort studies**													
	**The two groups were similar and recruited from the same population**	**The exposures measured similarly to assign people to both exposed and unexposed groups**		**The exposure was measured in a valid and reliable way**	**Confounding factors were identified**	**Strategies to deal with confounding factors were stated**	**Participants free of the outcome at the start of the study**	**Outcomes were measured in a valid and reliable way**		**Follow up time reported**	**Follow up was complete, and if not, were the reasons to loss to follow up described and explored**	**Incomplete follow up were addressed**	**Appropriate statistical analysis used**
Aranyavalai et al., 2020 [44]	△	△		△	❍	❍	△	△		△	❍	△	△

△- Low risk, ❍- Moderate risk, □- High risk

### Randomised controlled studies

The included RCTs tested the effectiveness of distinct dance programs, exercise, education and nutrition as fall prevention interventions[42, 47–52, 57, 61, 62, 65, 68–70]. These studies also included exercise programs such as the Otago exercise program, Tai Chi and Baduanjin [49, 61, 65, 68, 70]. The Otago home-based exercise program is an evidence based fall prevention program which consists of balance and strength training exercises [49, 61, 68]. Tai Chi and Baduanjin are traditional Chinese mind-body exercises which include slow, continuous, flowing movements, coordinated with deep breathing and mental focus [65, 70].

Nine RCTs included ‘falls’ as an outcome. Exercise programs such as Baduanjin for 16 weeks, the Otago home-based exercise programme for six months, supervised balance, strength and gait training for 12 months, as well as patient education for either 12 weeks or 18 months showed statistically significant effect on preventing falls [42, 49, 52, 69, 70]. However, other studies with a 12 month home-based exercise program with lower extremities strengthening, stretching and balance training program, a 12 month moderate intensity resistance training program, daily micro-nutrient supplement for 24 months, and 8 week group based physical and cognitive training 12 months on falls, did not have any effect on falls [47, 48, 50, 57].

‘Falls risk’ was included as an outcome in an RCT [65]. The Physiological Profile Assessment (PPA) was the tool used to assess fall risk. From the results of this study Tai Chi for 15 weeks was effective in reducing fall risk [65].

The Otago home-based exercise program for six months, 12 weeks senior dance program, 12 weeks Thai traditional dance, 16 weeks Baduanjin exercise and 12 months supervised balance, strength and gait training programs were effective in improving balance in older adults [42, 49, 51, 62, 70]. However, a 12 months home-based exercise program with lower extremities strengthening, stretching and balance training, did not improve balance [47].

Similarly, FOF significantly reduced with 8 weeks Otago exercise programs, 8 weeks chair squat exercise, 12 months supervised balance, strength and gait training program and 12 weeks fall prevention education [42, 52, 61]. However, in other studies 12 months of exercise interventions including balance training, strength training, stretching, 12 weeks of senior dance and 8 weeks group based physical and cognitive training did not significantly reduce FOF [47, 51, 57].

Mobility was improved with interventions such as the Otago exercise program, senior dance program, Thai traditional dance, supervised balance, strength and gait training program, micronutrient supplement with physical activity and group based physical cognitive training [42, 49–51, 57, 62]. Yet, a 12 month home-based exercise program with lower extremities strengthening, stretching and balance training did not show an effect [47].

When considering the two studies which used dance as an intervention, results indicated that either senior dance or Thai traditional dance could significantly improve balance and mobility among older adults [51, 62]. In contrast, a multifactorial intervention for 12 weeks did not show any effect on fall rate or recurrence of falls [68].

### Quasi-experimental studies

Quasi-experimental studies were undertaken using different experiment groups; therefore, meta-analysis was not possible except for the two studies which were pooled together to assess the effect of exercise on falls [45, 46, 53, 63, 67]. A health education program was effective in significantly reducing falls in 12 months [46].

Exercises as standalone intervention for four and eight weeks, were effective in significantly improving mobility, balance and gait speed while reducing fall risk among older adults [53, 67].

Russian stimulation, a type of electrical muscle stimulation, combined with isometric strengthening for 12 weeks significantly improved balance, strength, and mobility [63]. Anticipatory postural adjustments focused training for 5 months, was also effective in improving balance control and quality of life [45].

### Other studies

A cohort study found that older adults who walked about 5000 steps a day showed significantly lower incidence of falls when compared to a group of older adults who took fewer steps. However, as this was an observational study, causality cannot be inferred [44]. A cross-sectional study demonstrated that FOF was significantly lower among older adults who participated in regular physical activity when compared to those who did not, but the direction of this relationship is unclear due to the study design [64].

### Overall summary of narrative synthesis

In summary, the findings from this narrative synthesis highlight the variety of interventions evaluated to prevent falls among community-dwelling older adults in LMICs. While exercise programs, senior dance, Thai traditional dance and education interventions showed a positive effect on certain outcomes such as reducing falls and fall risk in some studies, their impact was inconsistent across others. Among the various exercise interventions, balance training and structured exercise programs demonstrated the most positive effects on fall prevention and related outcomes.

## Discussion

This systematic review assessed the effectiveness of interventions designed to prevent falls among community-dwelling older adults living in LMICs. Our findings identified mixed results in terms of the effectiveness of different interventions and the effect on different fall-related outcomes.

The meta-analyses conducted in this study showed that exercises improved balance in community dwelling older adults living in LMICs, but it did not confirm any effect of identified interventions on fall rate or other fall related outcomes among community dwelling older adults living in the LMICs. Although the effect of exercise programs on number of falls, fear of falling, balance and mobility were tested with meta-analyses, the results did not show any statistically significant effects, except for balance. This is in contrast to the strong research evidence from large systematic reviews, dominated by large studies in HIC. The small number of participants in the studies in our review may have reduced the statistical power of the meta-analysis, which could explain the absence of statistically significant intervention effects. In contrast, the narrative synthesis provided certain insights regarding the potential effect of specific fall prevention interventions observed in LMICs.

The narrative synthesis found that exercise interventions, senior dance program, Thai traditional dance and education programs were among the interventions used to prevent falls among community-dwelling older adults living in the LMICs. The exercise interventions utilised multimodal exercises including balance training, and Tai Chi more commonly.

To date, there have been many studies that have looked at the effect of falls prevention interventions on older people living in the community [17, 74, 75]. A 2019 systematic review provides strong evidence that exercise is effective in preventing falls [17]. These studies, however, have been conducted mostly in high income countries and the current review is the first to review the effect of fall prevention interventions on older people living in LMICs. It is important to assess the effectiveness of fall prevention interventions in LMICs because the World Health Organization states that 80% of fall related deaths occur in LMICs. Limited access to preventive health services, lower awareness about fall risk, inadequate environmental safety, malnutrition, increasing prevalence of disease conditions, fewer opportunities for structured exercise programs, economic barriers in accessing health care can impact the effect of fall prevention interventions for community dwelling older adults in LMICs when compared to HICs [9, 76–82]. Given that very few of the 108 studies in the 2019 review were conducted in LMIC, that many LMIC are among the fastest ageing populations, and that there are several factors that may limit the direct translation of fall prevention interventions from high income countries to LMIC, this study focussing on LMIC is important [17]. The 2019 systematic review concludes that exercise has a significant effect on falls and can be used as a standalone intervention for preventing falls among community-dwelling older adults [17]. Another systematic review states that balance is significantly improved with exercise especially with exercise that challenge balance [75]. However, the meta-analyses from our review did not identify any significant effect of exercises or any other interventions on preventing falls or improving any fall-related interventions in LMICs, except for balance. On the other hand, based on the narrative synthesis of single studies not included in the meta-analyses, exercise programs, dance interventions such as senior dance and Thai traditional dance, and mind body exercises such as Tai chi and Baduanjin provided promising findings in significantly improving functional ability of older adults and reducing fall-related risk factors as standalone interventions [51, 56, 59, 62, 65]. Multiple-component interventions such as ‘education plus dance’, and ‘education and environmental’ modifications were also effective in improving fall-related outcomes such as balance and mobility. Hence, this can be considered as promising evidence for practice on fall prevention for community-dwelling older adults in LMICs.

The discrepancy between the previous systematic review and the current review could have been due to various reasons [17]. The 2019 systematic review included studies from both high-income countries and LMICs, while most of the studies were from high-income countries. The current review included studies only from LMICs. The effect of fall prevention interventions in LMICs could have been masked by the results from high-income countries. There could have been other factors such as a smaller number of studies, differences in LMICs such as lack of quality interventions, poor economic support, lack of resources, lack of awareness, cultural differences, lifestyle, family support, environmental differences, and type of footwear, which may have contributed to this discrepancy.

There were several limitations faced by the research team which could have impacted the results of this review. Only nine RCTS were eligible to be included for the meta-analysis. The small sample size of the included studies reduces the robustness of the evidence identified. Since, the search was limited to English language, publications in other languages might have been missed in this systematic review. This review includes studies from only 11 LMICs which may affect the generalizability of this study to all LMICs. The search and review target outcomes were limited to some most frequently reported fall risk factors and fall related measures. Future reviews may explore a more extensive range of fall risk factor outcomes.

Future RCTs with larger sample sizes and robust design should be considered to provide clearer evidence about the effect of fall prevention interventions in LMICs. Use of mixed methods designs may also help to understand the unique facilitators and barriers for successful implementation in LMIC. It is also recommended to conduct future reviews including studies which are published in languages other than English. 

This review concludes that a number of interventions including senior dance programs, Thai traditional dance, education programs and multimodal exercises incorporating balance training and Tai Chi, were identified as fall prevention approaches used in LMICs in the included studies. The meta-analysis showed that exercises improved balance in community-dwelling older adults living in the LMICs. However, it is important to note that the evidence supporting the efficacy of these interventions in reducing falls or other fall-related outcomes is inconclusive based on the studies included in this review. Further adequately powered high-quality trials are needed to validate these findings and develop tailored fall prevention strategies for implementation in LMICs. 

## Supplementary Information

Supplementary Material 1.

## Data Availability

The included studies were retrieved from Medline, Embase, Cochrane, Scopus and CINAHL. All data used and analysed in this study are presented within this article.

## Abbreviations

FOF: Fear of falling
FESI: Falls efficacy scale international
GNI: Gross national income
LMIC: Low- and middle-income country
OECD: Organization for economic co-operation and development
ProFaNE: Prevention of falls network europe
PROSPERO: International prospective register of systematic reviews
RCT: Randomised controlled trial
ROB: Risk of bias
SMD: Standardised mean difference
TUG: Timed-up and go
USD: United states dollar
